# Alpha and Theta Oscillations Are Causally Linked to Interference Inhibition: Evidence from High-Definition Transcranial Alternating Current Stimulation

**DOI:** 10.3390/brainsci13071026

**Published:** 2023-07-04

**Authors:** Yan Zhu, Di Wu, Kewei Sun, Xianglong Chen, Yifan Wang, Yang He, Wei Xiao

**Affiliations:** Department of Military Medical Psychology, Air Force Medical University, Xi’an 710032, China; 13898277961@163.com (Y.Z.); wudi0426@outlook.com (D.W.); xlxsunkewei@126.com (K.S.); qq17687658@163.com (X.C.); wyff_1220@163.com (Y.W.); hy6548182902022@163.com (Y.H.)

**Keywords:** inhibitory control, interference inhibition, response inhibition, lDLPFC, HD-tACS, neural oscillations, color-word Stroop task, Go/NoGo task

## Abstract

(1) Background: The Go/NoGo task and color-word Stroop task were used to investigate the effect of applying different frequency bands of neural oscillations to the lDLPFC on inhibitory control modulation. (2) Methods: Participants were randomly categorized into four groups and received HD-tACS at 6, 10, and 20 Hz or sham stimulation at 1.5 mA for 20 min. All participants performed a color-word Stroop task and Go/NoGo task before and immediately after the stimulation; closed-eye resting-state EEG signals were acquired for 3 min before and after the tasks. (3) Results: There were no significant differences in the Go/NoGo behavioral indices task across the four groups. In the color-word Stroop task, the Stroop effect of response time was significantly reduced by 6 and 10 Hz stimulations compared to sham stimulation, and the Stroop effect of accuracy was significantly reduced by 10 Hz stimulation. There were no significant differences in the frequency range-specific (delta, theta, alpha, beta, or gamma) resting EEG power before and after stimulation. (4) Conclusions: HD-tACS at 6 and 10 Hz effectively improved participants’ performance on the color-word Stroop task, demonstrating the importance of the lDLPFC in interference inhibition and supporting a causal relationship between theta and alpha oscillations in interference inhibition.

## 1. Introduction

Inhibitory control is the cognitive process by which the brain suppresses or inhibits the input, response output, or internal processing of external stimuli that are not relevant to the current task when processing information, and it is an important component of an individual’s executive function and self-control [1,2]. Inhibitory control is the foundation of accurate performance [3] and is vital for flexible and goal-directed behavior [4]. It has been shown that inhibitory control is important for memory retrieval [5]; motor processing [6]; mathematical and reading achievements [7,8,9]; and even higher-level cognitive processes, such as reasoning ability [10] or decision making [11,12].

Inhibitory control can be divided into the components of interference inhibition and response inhibition [13,14]. Response inhibition, which refers to the ability to inhibit behavioral responses that do not meet current needs or are inappropriate, is a late component of inhibitory control. Interference inhibition, which refers to the ability to inhibit interference from competing stimuli, is an early component of inhibitory control [15]. In studies of inhibitory control, researchers have designed multiple experimental paradigms to measure individual inhibitory control; for interference inhibition, the corner-word Stroop task has been used primarily, whereas for response inhibition, the Go/NoGo task has been used primarily [16].

Researchers have used functional magnetic resonance imaging (fMRI) and electroencephalography (EEG) techniques to identify the “cognitive control network,” which is the coordinated activation of multiple brain regions during inhibitory control. These brain regions include the dorsolateral prefrontal cortex (DLPFC), medial frontal cortex (MFC, including the parietal cortex and anterior cingulate cortex), motor areas, and cerebellum [17,18,19]. Although inhibitory control tasks involve many brain regions, scholars have used neuroimaging studies to highlight the importance of the DLPFC in inhibitory control processes [20,21,22,23,24,25]. Researchers have generally considered that the function of the DLPFC is to enable individuals to select appropriate responses, inhibit inappropriate ones, and control impulsive behavior in concert with regions of the brainstem and basal ganglia (i.e., the impulse system) [26,27]. Some brain injury studies have shown that patients with DLPFC impairment have lower behavioral performance than normal controls when performing inhibitory control tasks, which also indicates the importance of the DLPFC for inhibitory control [28,29].

At the same time, researchers have found that the left DLPFC (lDLPFC) plays a more important role in inhibitory control than the right DLPFC (rDLPFC). A study using the cognitive reflection test found that anodal tDCS of the lDLPFC significantly improved behavioral performance in the task [20]. Nevertheless, another tDCS study found that stimulation over the right DLPFC did not modulate delayed response inhibition performance [30]. The authors argued that the result might have been due to differences between the roles of the left and right DLPFC in inhibitory control. A neuroimaging study that discovered lDLPFC activation during the presentation of insight problems [31] further emphasized the lDLPFC’s specific function in insight problem solving. This activation was linked to the removal of unhelpful cognitive limitations and the ensuing resolution of mental blocks. The lDLPFC was also discovered to be preferentially activated by creative tasks as opposed to control noncreative tasks [32]. This is likely because of the DLPFC’s involvement in the top-down organization of the creative process, which involves a variety of functions mediated by a distributed network in which this region seems to play a key role.

Studies have shown that synergistic integration between multiple brain regions is achieved through neural oscillations [33]. This integration can exist in many functional domains, each with a different frequency [34,35]. Previous electrophysiological studies have found that in Go/NoGo tasks, individuals produce greater midfrontal theta oscillations, corresponding to the midfrontal N2 component, when response inhibition occurs successfully [36]. This outcome is also consistent with the results of color-word Stroop task EEG studies [37,38,39]. This oscillatory activity in the midfrontal theta band (4~7 Hz) may reflect a neural mechanism of conflict detection [40]. It has also been suggested that theta oscillations are associated with a variety of cognitive processing tasks and may be an indicator of cognitive effort and the realization of the need for top-down control [41]. Moreover, it has been shown that alpha oscillations are involved in inhibitory processes and contribute to various cognitive processes, such as attention and memory [42]. Alpha oscillation underactivity or increased slow-wave activity has been associated with cognitive deficits and a lack of inhibitory control [34]. Furthermore, beta oscillations are considered a classical indicator of action response readiness [43], and desynchronization of beta oscillations is closely related to inhibitory control [44]. One study found that successful NoGo trials produced obvious desynchronization of the beta oscillations [45]. In summary, studying the causal relationship among theta, alpha, and beta oscillations of the left DLPFC (lDLPFC) and inhibitory control then becomes the focus of further investigation into the neurophysiological mechanisms of inhibitory control.

To demonstrate a causal relationship between neural oscillations and inhibitory control in the lDLPFC, the transcranial alternating current technique (tACS) has received increasing attention within the last decade. The advantage of transcranial alternating current stimulation (tACS) as a noninvasive brain stimulation (NIBS) technique is that it allows us to causally infer the characteristics of neural oscillations [46]. The membrane potentials of many neurons are rhythmically and simultaneously altered by the injection of alternating current into the brain [47]. This effectively entrains networks exogenously [48]. Brain oscillations can be causally linked to changes in cognitive functions by regulating rhythmic brain activity. Furthermore, numerous studies have demonstrated that tACS can influence common cognitive functions. [49]. For example, Pahor et al. [50] found that theta-range tACS of the lDLPFC improved performance on simple items of a problem-solving test by altering the attentional component. Furthermore, Yaple et al. [51] found that theta-range tACS of the bilateral DLPFC during decision making that required cognitive control reduced risky behavior in decision making, which confirmed that the DLFPC is a critical region for adaptation in decision-making strategies. Likewise, these results suggest that the DLPFC is a promising target for the imposition of tACS during cognition.

In summary, with a high-definition (HD) electrode montage over the lDLPFC, we used tACS in the theta range (6 Hz), alpha range (10 Hz), and beta range (20 Hz). Our aim was to externally modulate the relevant band power of the lDLPFC and thereby causally change the function of the cognitive control network. The present study was designed to investigate how tACS-lDLPFC affects inhibitory control across frequency bands by evaluating different inhibitory control tasks, such as the Go/NoGo task and the color-word Stroop task. Moreover, to test whether tACS could lead to changes in endogenous brain rhythm [52], we recorded resting-state EEG signals to measure power differences in each frequency band (delta, theta, alpha, beta, and gamma) before and after stimulation.

## 2. Materials and Methods

### 2.1. Participants

Sixty undergraduate students (28 males and 32 females, mean age 23.02 ± 2.49 years from a military medical university were recruited to participate in this study through the internet and posters. We used questionnaires to ensure that all participants were right-handed individuals with normal or corrected-to-normal vision and did not have any history of mental or neurological diseases or any tACS-related contraindications. To ensure an even gender distribution among groups, participants of different genders were separated, and participants within each gender were randomly divided into four groups: 6 Hz stimulation (*n* = 15), 10 Hz stimulation (*n* = 15), 20 Hz stimulation (*n* = 15), and sham stimulation (*n* = 15). This randomization protocol ensured that the ratio of men to women was the same in all groups. There were no significant differences in mean age (*p* > 0.05). All participants were required not to stay up late before the experiment and not to drink or eat coffee, alcohol, tea, and other stimulating drinks and food.

All participants volunteered to partake in the experiment. Written informed consent was obtained from the participants, and a certain remuneration was paid to the participants after the experiment. This study was conducted in accordance with the Declaration of Helsinki and approved by the Ethics Review Committee of Xijing Hosp.

### 2.2. Experimental Protocol

**Figure 1:**
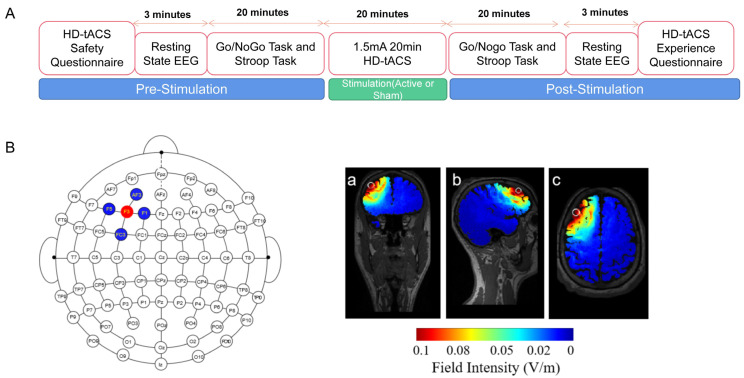
Experimental design. (**A**) Design of the experiment. The lDLPFC was stimulated using HD-tDCS with a current intensity of 1.5 mA and for a duration of 20 min. The Go/NoGo task and color-word Stroop task were administered pre, during-, and poststimulation, and EEG data were collected throughout the experiment. (**B**) A 4 × 1 ring configuration was used in which the target electrode was placed at the F3 location, while return electrodes were placed at F5, F1, FC3, and AF3. The electric field was stimulated using SIMNIBS. (**a**) Coronal section. (**b**) Median sagittal section. (**c**) Transverse section.

The experiment had a single-blinded, sham-controlled design and was conducted in a soundproof, low-light, electromagnetic-shielded laboratory. On the day of the experiment, the experimental protocol was explained once to the participants, who then signed a consent form and completed a tACS questionnaire. The questionnaire investigated the participants’ eligibility for the study and inquired if they had consumed any psycho-stimulants before the experiment. Closed-eye resting-state EEG signals were acquired before and after stimulation of the lDLPFC (F3 locus), the Go/NoGo task and the color-word Stroop task were completed, and the order of the two tasks was balanced. The design of the experiment is shown in Figure 1A. After the experiment, a tACS experience questionnaire inquired if the participants felt any side effects of stimulation, along with mild headache or minor discomfort, scalp pain, neck pain, burning sensation, tingling, and itching.

### 2.3. HD-tACS

The 4 × 1 multichannel stimulation device (Model 4 × 1-C3A, Soterix Medical Inc., Woodbridge Township, NJ, USA) was conducted through a battery-powered HD-tACS stimulator (Model 1300A, Soterix Medical Inc.). As seen in Figure 1B, during active HD-tACS sessions, the bipolar sinusoidal current was delivered over the lDLPFC at a frequency of either 6, 10, or 20 Hz. The current was ramped up to stimulation intensity over 30 s at the beginning of the stimulation, maintained during the middle of stimulation, and ramped down to 0 mA over 30 s at the end of stimulation. The current was raised to the simulation strength for sham stimulation over 30 s and then ramped down to 0 mA over 30 s at the beginning. Similarly, the current increased to the stimulation intensity over 30 s and then ramped down to 0 mA over 30 s at the conclusion. Throughout and in the midst of the sham stimulation, the current was held at 0 mA.

A current flow model produced with the aid of HD-Explore software (Soterix Medical Inc.) was used to establish an electrode montage to identify the target site. The central electrode was positioned at F3 in accordance with the International 10-10 EEG standard, and four return electrodes were positioned at F5, F1, FC3, and AF3 [53] (see Figure 1B). To ensure that the impedance values of each electrode were ≤5 kΩ throughout the stimulation, five Ag/AgCl electrodes (outer diameter: 12 mm, inner diameter: 6 mm; Soterix Medical Inc.) were inserted into plastic electrode holders (Soterix Medical Inc.) filled with conductive gel. The theoretical current density of this electrode montage with a 1.5 mA stimulation intensity is also shown in Figure 1B. At the end of the experiment, all participants were questioned about whether they could detect the presence of stimulation.

### 2.4. Go/NoGo Task

**Figure 2:**
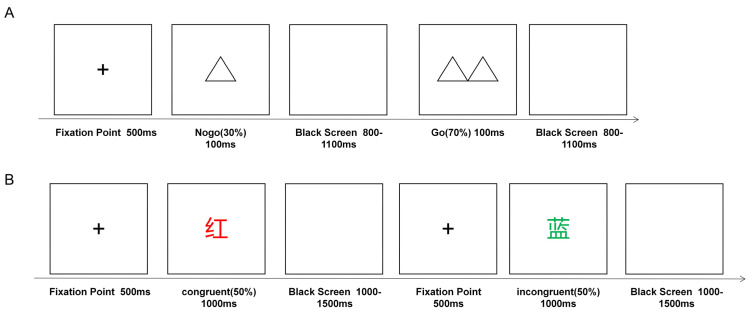
Tasks schematic. (**A**) Go/NoGo task. (**B**) Color-word Stroop task.

The Go/NoGo task was used to examine the ability of active inhibition in response inhibition. The task consisted of 2 blocks, each with 100 trials, divided into 2 types of double triangles (“go”) and single triangles (“nogo”). During the task, two types of stimulation were presented randomly in the center of the screen in a 7:3 ratio, with a presentation time of 100 ms and an interval of 800–1100 ms. Participants were asked to quickly press the left key on the keyboard when a double triangle appeared and not to respond when a single triangle appeared. Participants were allowed to rest between each block and then press any key to continue the task when they were ready. The average response time (RT) and accuracy of NoGo trials (i.e., correct rejection rate) were calculated to assess response inhibition. The shorter the RT, the higher the correct rejection rate, and the better the subject’s ability to inhibit their responses (see Figure 2A).

### 2.5. Color-Word Stroop Task

The color-word Stroop task was used to examine interference inhibition, and the stimuli were four capitalized Chinese words for colors (green, red, yellow, and blue) presented with matching or different font colors. The font size was consistent and uniformly used with a black background. The task required the participants to choose the actual color of the font (“red,” “blue,” “green,” or “yellow”) according to the corresponding F, G, J, and K keys and to use the left middle finger, left index finger, right index finger, and right middle finger, respectively, to press the keys. During the experiment, the target stimulus was presented for 1000 ms after a 500 ms fixation point, followed by the presentation of a blank screen for 1000–1500 ms; the participants had up to 1800 ms to respond. There was a short break halfway through the task, and participants continued the task by pressing any key at the end of the break. The total number of trials in this experiment was 240, 120 of which were in the consistent condition (e.g., “红” RED written in red) and 120 of which were in the inconsistent condition (e.g., “蓝” BLUE written in green) (see Figure 2B). The Stroop effects of RT (RT difference between congruent and incongruent trials) and accuracy (ACC, ACC difference between congruent and incongruent trials) were measured to assess interference inhibition, and the smaller the Stroop effect, the better the interference inhibition ability (see Figure 2B).

### 2.6. Resting-State EEG Recording and Preprocessing

Before and after the application of tACS, EEG signals were captured during a 3-min resting state with the eyes closed using a sampling frequency of 1000 Hz. Channel activity was referenced online to Fz, and the impedance was kept below 5 kΩ. The EEG signals were captured using the 10-10 system from 32 electrodes that were connected to an electrocap (Brain Products, BrainAmp MR Plus). Participants were instructed to close their eyes and unwind in a dimly lit room free of any outside noise throughout the recording of the resting-state EEG signals.

### 2.7. Data Analysis: Behavioral Data

First, we calculated the RT and correct rejection rate as the behavioral outcome of the Go/NoGo task and the Stroop effect of RT and ACC as the behavioral outcome of the color-word Stroop task. Second, to ensure that the data for all participants followed a normal distribution, all the data were screened to eliminate data that deviated by more than three standard deviations from the mean. Finally, when comparing the behavioral outcomes (RT, correct rejection rate, and Stroop effect) with two factors, time (pre vs. post) and group (6 Hz vs. 10 Hz vs. 20 Hz vs. Sham), repeated-measures analysis of variance (ANOVA) was primarily used to analyze the behavioral data using the statistical program SPSS 22.0.

### 2.8. Data analysis: Resting-State EEG Data

Preprocessing was carried out on resting-state EEG data collected with the eyes closed using EEGLAB and a specially created MATLAB script (MathWorks, Natick, MA, USA). With a sampling rate of 1000 Hz and a whole-brain averaging reference, continuous EEG data were concave filtered at 48–52 Hz and bandpass filtered between 0.1 and 40 Hz using an FIR filter. The EEG data were split into 2-s chunks. Ocular artifacts were removed using the algorithm reported by Gratton, Coles, and Donchin (1983) [54]. Topographical interpolation was employed to calculate new values for bad channels, with a maximum of three per participant (data were excluded if the individual had more than three bad channels) [55]. Independent component analysis (ICA) [56] was used to eliminate eye movement and blink artifacts from the remaining EEG samples. Extreme values greater than 100 V were finally eliminated. FFT analysis was used to derive power spectrum metrics (dB) from the preprocessed data. Finally, individual power values were averaged for each participant over the frequency range of interest, and prestimulation and poststimulation power values were calculated separately.

The average of power values retrieved in the frequency range between 4 and 7 Hz is referred to as “theta power” moving forward. In a similar vein, we use the term “alpha power” to denote the mean of power levels obtained in the 8~12 Hz frequency range. The average of power values retrieved in the frequency range between 15 and 25 Hz is referred to as “beta power” in this context. Paired samples t-tests were used to compare the theta, alpha, and beta power differences at channel F3 before and after HD-tACS.

## 3. Results

### 3.1. Demographic and Descriptive Information

Data from a total of 60 valid cases were entered for statistical analysis, including those of patients who received 6 Hz (*n* = 15), 10 Hz (*n* = 15), 20 Hz (*n* = 15), and sham (*n* = 15) stimulation. There was no significant difference between the ages of the participants in the three experimental groups and the one sham group (*F* = 0.787, *p* = 0.506). None of the participants reported the presence of retinal phosphenes with any active HD-tACS. Moreover, only five participants reported a mild skin sensation that disappeared after a few seconds of stimulation. None of the other participants reported skin sensations.

### 3.2. Behavioral Results

We conducted a one-way ANOVA on the prestimulation data for the correct rejection response of the Go/NoGo task and the Stroop effect of the color-word Stroop task, with a group (6 Hz, 10 Hz, 20 Hz, and sham) as the between-participants factor. There were no significant differences in baseline performance for the correct rejection rate or (*F* < 1) for the Stroop effect of RT (*F* < 1), indicating the success of randomized grouping.

#### 3.2.1. Effects of HD-tACS on the Go/NoGo Task

We calculated the average RT and the correct rejection rate in the Go/NoGo task and used them as indicators of suppressed response performance in the Go/NoGo task with a two-factor (time vs. group) repeated-measures ANOVA.

**Figure 3:**
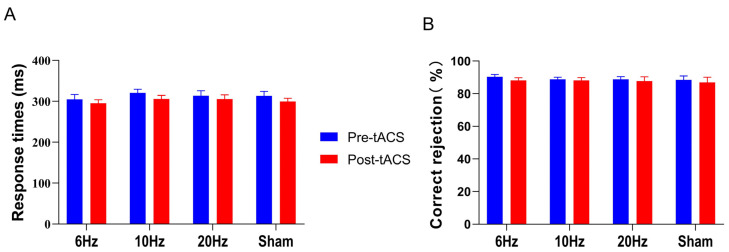
In the Go/NoGo task, means and standard errors for (**A**) response time and (**B**) correct rejection rate (%) showed no significant changes among groups. All the data are presented as the mean ± S.E (*n* = 60).

ANOVA results for RT showed a significant time main effect (*F*_(1,56)_ = 25.364, *p* < 0.001, *η^2^* = 0.312), a nonsignificant group main effect (*F _(3,56)_* = 0.330, *p* = 0.804, *η^2^* = 0.017), and a nonsignificant interaction (*F _(3,56)_* = 0.456, *p* = 0.714, *η^2^* = 0.024), as shown in Figure 3A. The results of the ANOVA on the correct rejection rate revealed that the time main effect (*F _(1,56)_* = 2.285, *p* = 0.136, *η^2^* = 0.039), the group main effect (*F _(3,56)_* = 0.113, *p* = 0.952, *η^2^* = 0.006), and the interaction effect between the two (*F _(3,56)_* = 0.130, *p* = 0.942, *η^2^* = 0.007) were not significant, as shown in Figure 3B.

#### 3.2.2. Effects of HD-tACS on the Color-Word Stroop Task

RT (ms) and ACC (%) were calculated for both congruent and incongruent congruency in the color-word Stroop task, and the Stroop effects on RT and ACC were calculated and used as an indicator of performance in the color-word Stroop task with a two-factor (time vs. group) repeated-measures ANOVA.

A significant difference in RT was observed between congruency (congruent vs. incongruent) prior to stimulation (*t* = −17.244, *p* < 0.001). Participants had longer RTs in incongruent trials (698.07 ± 119.41 ms) than in congruent trials (597.99 ± 85.70 ms). The difference in ACC between congruency (congruent vs. incongruent) was significant (*t* = 5.645, *p* < 0.001). Participants had higher ACC in the congruent trials (96.60 ± 2.84%) than in the incongruent trials (92.92 ± 5.99%).

**Figure 4:**
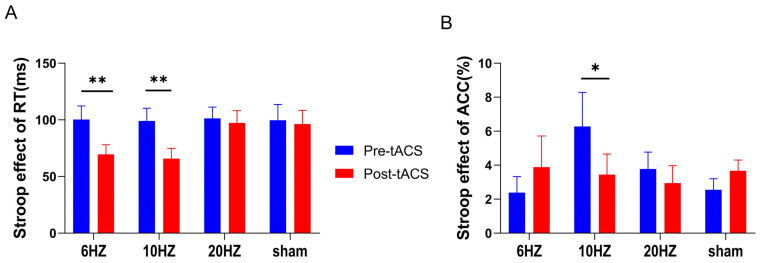
In the color-word Stroop task, means and standard errors for Stroop effects on RT and ACC. (**A**) shows that the Stroop effect on RT was significantly lower in the 6 Hz and 10 Hz groups relative to that in the sham stimulation group after stimulation. (**B**) shows that the Stroop effect on ACC in the 10 Hz group was significantly lower after stimulation than that in the sham stimulation group. All the data are presented as the mean ± S.E (*n* = 60); * *p* < 0.05, ** *p* < 0.01.

The Stroop effects on RT and ACC were calculated and used as dependent variables in a two-factor (time vs. group) repeated-measures ANOVA. The results showed a significant main effect of time for the Stroop effect on RT (*F*_(1,56)_ = 13.700, *p* < 0.001, *η*^2^ = 0.197) and a significant interaction effect of group and time (*F*_(3,56)_ = 2.866, *p* = 0.045, *η*^2^ = 0.133). Simple effects tests revealed a significantly lower Stroop effect on RT for the 6 Hz and 10 Hz group posttests compared to that in the sham stimulation group (*p* < 0.01). There was no significant difference in the Stroop effect on RT for the 20 Hz group (see Figure 4A).

The time main effect (*F*_(1,56)_ = 0.369, *p* = 0.546, *η*^2^ = 0.007) and the group main effect (*F*_(3,56)_ = 0.567, *p* = 0.639, *η*^2^ = 0.029) of the Stroop effect on ACC were not significant, but the interaction effect between group and time was significant (*F*_(3,56)_ = 3.489, *p* = 0.021, *η*^2^ = 0.157). Simple effects tests showed that only the 10 Hz group had a significantly lower Stroop effect on ACC (*p* = 0.01) (see Figure 4B).

### 3.3. Effects of HD-tACS on EEG Activity

**Figure 5:**
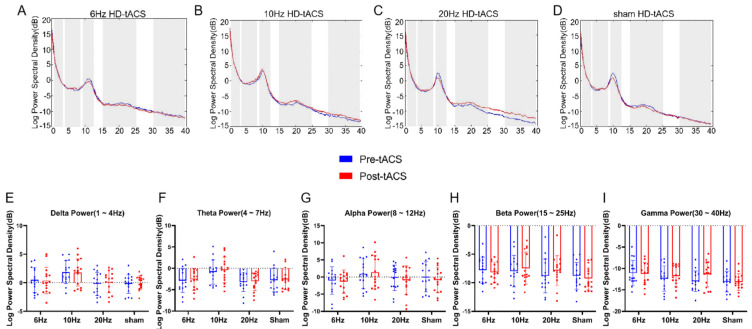
Effects of HD-tACS on resting-state EEG oscillations show no significant changes. Power spectrum of resting-state EEG signals before and after 6 Hz (**A**), 10 Hz (**B**), 20 Hz (**C**), or sham (**D**) HD-tACS stimulation from channel F3. The frequency bands of interest (delta, theta, alpha, beta, and gamma) are highlighted by the shaded gray areas. The average power values obtained from these power spectra are shown in the plots below as a function of the type of group (i.e., tACS condition) and time (pre- vs. posttACS), separately for the delta (**E**), theta (**F**), alpha (**G**), beta (**H**) and gamma (**I**) frequency bands. Dots represent individual values. Error bars indicate standard error of the mean (SEM). All the data are presented as the mean ± S.E (*n* = 60).

By comparing the power of the oscillations associated with the closed-eye resting-state EEG signals before and after the F3 channel stimulation, we observed no significant power changes in the theta (4~7 Hz), alpha (8~12 Hz), and beta (15~25 Hz) bands. We also analyzed delta (1~4 Hz) and low gamma (30~40 Hz), though we did not find any significant power changes in these bands (all *ps* > 0.26) (see Figure 5).

## 4. Discussion

In this study, we investigated the role of theta, alpha, and beta oscillations in interference inhibition and response inhibition by using HD-tACS with a current of 1.5 mA for 20 min as a way to introduce exogenous neural oscillations into the lDLPFC. We used the color-word Stroop task and the Go/NoGo task, as well as resting-state EEG, to study stimulation-induced behavioral changes and their endogenous neural changes. Our aim was to enrich our understanding of the neural mechanisms of inhibitory control and to explore the causal role of prefrontal neural oscillations in the modulation of inhibitory control.

According to our research, under the color-word Stroop task, theta-range tACS significantly reduced the Stroop effects on RT compared to sham stimuli. This result was consistent with the results of Lehr et al. [57], who found that theta-range tACS both reduced the Stroop effect on RT and modulated the adaptive mechanisms of the cognitive control network after performing theta-range tACS on the lDLPFC. Klírová et al. [58] found that personalized HD-tACS of the medial prefrontal cortex (MFC) resulted in increased interference scores in the Stroop task, while another study using different frequency bands of tACS reported that theta-range tACS improved the speed of responses to consistent stimulation after incorrect execution [59]. Thus, it is plausible that theta-range tACS elicits sustained neural activity in the prefrontal regions of the brain, which further reflects the modulation of behavioral performance. Our findings also further confirm the findings of previous studies indicating that there is a causal relationship between prefrontal oscillations in the theta band and conflict processing [57,60].

However, unlike these studies that found that the theta band specifically modulates interference inhibition, we found that the alpha band also significantly improved behavioral performance in the color-word Stroop task. HD-tACS in the alpha band significantly reduced not only the Stroop effect on RT but also the Stroop effect on ACC. We believe there are several possible reasons for this outcome. First, according to previous studies, alpha-range tACS can enhance visual perception [61], numerosity discrimination [62], attention [63,64], and other abilities, and improvement of all these abilities may affect interference inhibition task performance. Second, it has been demonstrated that alpha-range tACS affects other EEG frequencies, i.e., cross-frequency interaction (CFI) [65]. For example, Castellano et al. [66] found that the amplitudes of both alpha and gamma bands increased during 20 min of 10 Hz tACS in the occipital region. Helfrich et al. [67] found that 1 mA applied in the Oz-Cz region for 20 min of 10 Hz tACS could be observed in the alpha-gamma phase-amplitude CFI. Therefore, we conjecture that CFI effects may be generated by other frequency bands after the exogenous introduction of alpha oscillations, which may have an impact on the suppression control capability. However, further studies are needed to verify this conjecture. Third, conflict detection is controlled by the theta band of the dACC located in the MPC and DLPFC. However, the modulation of conflict detection by the dACC also requires information transfer between different brain regions. Information transfer between the LPFC and posterior brain regions and between the LPFC and sensorimotor brain regions is facilitated by low-frequency (<14 Hz) phase synchronization in the anterior-posterior network, and this information exchange allows the prefrontal cortex to exert control over sensory processes in the lower layers. This control is associated with alpha oscillations in the ventral middle prefrontal lobe, where the vmPFC is located [68]. As shown above, exploring the role of different frequency bands in relevant brain regions also helps us to further construct inhibitory control-related cognitive control networks and further validate the results of this experiment.

In addition, we found no significant changes in behavioral indices in the Go/NoGo task before and after stimulation in each frequency band compared to that before and after sham stimulation. This result demonstrated that the lDLPFC plays a more important role in interference inhibition than in response inhibition. This result also correlated with previous fMRI results indicating that the response inhibition process in the Go/NoGo task mainly involves brain regions such as the inferior frontal gyrus (IFG) and supplementary motor areas (SMA/preSMA) [6,69], whereas the interference inhibition process in the color-word Stroop task mainly involves the anterior cingulate cortex and the DLPFC [70]. Daughters et al. [71] found that the application of alpha-range tACS in the bilateral DLPFC significantly increased the extent to which individuals discriminated and correctly responded to go trials and NoGo trials. We believe that the main reasons for the different results are as follows. First, unlike that study, which used a pair of standard sponge electrodes of 5 × 5 cm², we used five-ring Ag/AgCl HD electrodes. The advantage of HD electrodes is that they maintain the low intensity and safety characteristics of traditional electrodes while allowing for more precise targeting of the target cortex and more accurate and deeper electrical stimulation. In contrast, standard sponge electrodes can cause changes in the activity of many cortical areas (e.g., the DLPFC and preSMA) at the same time. Therefore, it is debatable whether the results reported by Daughters et al. were caused by the DLPFC. Second, analyzing the baseline level of the task, we found that the average correct rate of the pretest Go trials was greater than 95%, the average correct rate of the NoGo trials was higher than 88%, and the average RT was approximately 300 ms, which indicates that the task was relatively simple for the group of participants (college students); furthermore, these findings indicated that there was a ceiling effect, so the effect of one stimulation session was not reflected behaviorally. Therefore, in the next study, we will improve the Go/NoGo task by increasing the difficulty of the task, e.g., shortening the presentation time of the stimulation from 100 ms to 80 ms or shortening the interval between two stimuli from 800~1100 ms to 600~1000 ms.

Another aspect that needs to be mentioned is that we did not find significant changes in each frequency power of the resting-state EEG before and after stimulation. This result is similar to that in the study by Antal et al. [46]. Antal did not observe significant changes in any frequency band of EEG signals after tACS at 1, 10, 15, 30, and 45 Hz for each group of 8 to 16 participants. Antal suggested that the reasons for this result might be as follows: first, the small number of subjects and the low sample size were not statistically sufficient to produce significant results; second, the after-effects of tACS were weaker compared to those after tDCS, and a short single session of tACS might not be sufficient to change cortical excitability. Many experiments have shown that the after-effects of a single tACS session are maintained for approximately 20 min [72,73]. According to our experimental arrangement, there was approximately 30 min between the electrical stimulation and resting-state EEG recordings for the completion of the behavioral task, which may have exceeded the duration of the after-effects of the electrical stimulation in this experiment. Additionally, research has demonstrated that the pre- and poststimulation brain states affect whether tACS after-effects manifest. According to Ruhnau et al. [74], states with closed eyes do not result in an increase in phase coherence between stimulation and resting-state alpha. Neuling et al. [72] found that participants who had their eyes open during an EEG experiment with stimulation at IAF experienced a significant after-effect of increased alpha power, but participants who had their eyes closed did not. Neuling suggests that the alpha activity that occurs when the eyes are closed may be at a limit that cannot be increased, or conversely, the endogenous oscillation may be too strong to be affected by the tACS modest current. The present experiment obtained resting-state EEG data obtained in the closed-eye state. If we measured resting-state EEG with the participants’ eyes open, we might have obtained different results.

Another aspect of interest is that our study was conducted mainly in a group of college students in their 20s. Previous studies have shown that inhibitory control develops across the lifespan and relies on the same set of domain-general fronto-parietal networks [75]. Inhibitory control develops throughout childhood [76] and continues to mature during adolescence [77], then declines noticeably during normal aging. There are many studies on the causes of inhibitory control decline; the CRUNCH (Compensation-Related Utilization of Neural Circuits Hypothesis), first proposed by Rueter-Lorenz and Cappell (2008), suggests that older adults engage more brain regions than younger adults when performing the same cognitive task, in order to compensate for declining brain structure and function [78]. Moreover, studies have also shown that the effect of NIBS varies between age groups. Guerra et al. [79] found that the effect of gamma oscillations in boosting primary motor cortex plasticity is greater in young adults than in older adults. A possible reason is that the variability of NIBS effects in older adults may be elevated due to aging-related brain atrophy and increased intracranial CSF content, leading to a broadened current distribution [80].

Therefore, in our next study, we will use 6 Hz and 10 Hz tACS for multiple stimulations of the lDLPFC to further investigate the enhancement of inhibitory control by HD-tACS and the changes in the duration of the after-effects. Additionally, we will try to expand the age range of the participants and explore the effect of tACS-lDLPFC on inhibitory control in different age groups.

## 5. Conclusions

Our findings supported the notion that theta and alpha oscillations in the lDLPFC are causally related to the level of interference inhibition and that exogenous neural oscillations modulate inhibitory control. In the present study, we found that exogenous 6 Hz and 10 Hz HD-tACS applied to the lDLPFC cortex enhanced interference inhibition but did not change response inhibition. Our findings suggested that a specific brain region, i.e., the dorsolateral prefrontal cortex, has a more important role in conflict inhibition than in response inhibition.

## Data Availability

The datasets presented in this article are not readily available be- cause the datasets involve unfinished research projects. If necessary, requests to access the datasets should contact the corresponding author.
